# Honeysuckle Aqueous Extracts Induced *let-7a* Suppress EV71 Replication and Pathogenesis In Vitro and In Vivo and Is Predicted to Inhibit SARS-CoV-2

**DOI:** 10.3390/v13020308

**Published:** 2021-02-16

**Authors:** Ying-Ray Lee, Chia-Ming Chang, Yuan-Chieh Yeh, Chi-Ying F. Huang, Feng-Mao Lin, Juan-Ting Huang, Chang-Chi Hsieh, Jen-Ren Wang, Hsiao-Sheng Liu

**Affiliations:** 1 Department of Medical Research, Ditmanson Medical Foundation Chia-Yi Christian Hospital, Chiayi 600, Taiwan; yingray.lee@gmail.com; 2 Department of Microbiology and Immunology, School of Medicine, College of Medicine, Kaohsiung Medical University, Kaohsiung 807, Taiwan; 3 Department of Microbiology and Immunology, College of Medicine, National Cheng Kung University, Tainan 701, Taiwan; Chiaming0217@gmail.com; 4 Department of Traditional Chinese Medicine, Chang Gung Memorial Hospital, Keelung Medical Center, Keelung 204, Taiwan; b9005030@gmail.com; 5 Program in Molecular Medicine, School of Life Sciences, National Yang-Ming University, Taipei 112, Taiwan; bpshuang@gmail.com; 6 Institute of Biopharmaceutical Sciences, National Yang-Ming University, Taipei 112, Taiwan; 7 Department of Biochemistry, College of Medicine, Kaohsiung Medical University, Kaohsiung 807, Taiwan; 8 Institute of Bioinformatics and Systems Biology, National Chiao Tung University, Hsinchu 300, Taiwan; kiralintw@gmail.com; 9 Division of Big Data, Phalanx Biotech Group, Hsinchu 300, Taiwan; rachelhuang@phalanxbiotech.com; 10 Department of Animal Science and Biotechnology, Tunghai University, Taichung 407, Taiwan; cchsieh@thu.edu.tw; 11 Department of Medical Laboratory Science and Biotechnology, College of Medicine, National Cheng Kung University, Tainan 701, Taiwan; jrwang@mail.ncku.edu.tw; 12 Center for Cancer Research, College of Medicine, Kaohsiung Medical University, Kaohsiung 807, Taiwan; 13 M. Sc. Program in Tropical Medicine, College of Medicine, Kaohsiung Medical University, Kaohsiung 807, Taiwan

**Keywords:** honeysuckle, *let-7a*, EV71, virus replication

## Abstract

Honeysuckle (*Lonicera japonica* Thunb) is a traditional Chinese medicine (TCM) with an antipathogenic activity. MicroRNAs (miRNAs) are small non-coding RNA molecules that are ubiquitously expressed in cells. Endogenous miRNA may function as an innate response to block pathogen invasion. The miRNA expression profiles of both mice and humans after the ingestion of honeysuckle were obtained. Fifteen overexpressed miRNAs overlapped and were predicted to be capable of targeting three viruses: dengue virus (DENV), enterovirus 71 (EV71) and SARS-CoV-2. Among them, *let-7a* was examined to be capable of targeting the EV71 RNA genome by reporter assay and Western blotting. Moreover, honeysuckle-induced *let-7a* suppression of EV71 RNA and protein expression as well as viral replication were investigated both in vitro and in vivo. We demonstrated that *let-7a* targeted EV71 at the predicted sequences using luciferase reporter plasmids as well as two infectious replicons (pMP4-y-5 and pTOPO-4643). The suppression of EV71 replication and viral load was demonstrated in two cell lines by luciferase activity, RT-PCR, real-time PCR, Western blotting and plaque assay. Furthermore, EV71-infected suckling mice fed honeysuckle extract or inoculated with *let-7a* showed decreased clinical scores and a prolonged survival time accompanied with decreased viral RNA, protein expression and virus titer. The ingestion of honeysuckle attenuates EV71 replication and related pathogenesis partially through the upregulation of *let-7a* expression both in vitro and in vivo. Our previous report and the current findings imply that both honeysuckle and upregulated *let-7a* can execute a suppressive function against the replication of DENV and EV71. Taken together, this evidence indicates that honeysuckle can induce the expression of *let-7a* and that this miRNA as well as 11 other miRNAs have great potential to prevent and suppress EV71 replication.

## 1. Introduction

Honeysuckle (*Lonicera japonica* Thunb) is a traditional Chinese medicine (TCM). The aqueous extract of the flower bud can relieve fever and flu-like symptoms [1]. The active ingredients of honeysuckle flower extract include chlorogenic acid and isochlorogenic acid [1], which can suppress microbial activities both in vitro and in vivo. We have reported that ingestion of honeysuckle upregulates the innate microRNA (miRNA) *let-7a* to attenuate dengue virus-2 (DENV-2) replication and related pathogenesis both in vitro and in vivo [2]. In the present study, we further predicate that honeysuckle upregulated miRNAs including *let-7a* could target EV71 and SARS-CoV-2 according to our previous findings [2].

MicroRNAs (miRNAs) are small non-coding RNA molecules (containing about 22 nucleotides) involved in the regulation of many cellular functions and immune responses [3,4,5,6]. MiRNAs may regulate viral activity by targeting viral RNA genomes at the 5′UTR, 3′UTR or coding region of mRNA [6,7,8,9,10]. MiRNAs bind with specific targeting sites resulting in either degradation of the targeted RNA or blockage of translation. The highly permeable miRNAs in body fluid, tissues and organs have been used as biomarkers for disease diagnosis [11,12] and execute bioactivities located in cytoplasm, nucleus, mitochondria and even distant recipient cells by transportation through exosomes [10,13,14]. There is a growing interest in the use of miRNA-related immune responses to mitigate the effects of defective antibody production for the prevention or suppression of infectious diseases.

Enterovirus 71 (EV71) belongs to the enterovirus genus of the picornaviridae family and can be classified into three groups (A, B, C) and 11 genotypes (A, B1–5 and C1–5) [15,16]. EV71 contains a 7.5 kb single-stranded (+) RNA genome with a 5′untranslated region (UTR), a coding region, and 3′ UTR. Ribosomes travel from the 5′ end to the 3′ end of the RNA genome to synthesize a polyprotein in a cap-independent manner [17]. This polyprotein matures into 11 proteins including four capsid proteins (VP1–4) and seven non-structural proteins (2A, 2B, 2C, 3A, 3B, 3C, 3D) [18] through cleavage by viral proteases [19]. Before the viral genome RNA is encapsidated, VP0 is further cleaved into VP2 and VP4 by an autocatalytic mechanism [20]. EV71 patients generally show symptoms ranging from mild hand, foot and mouth disease (HFMD) to severe EV71-associated neurological complications. Although the symptoms of HFMD can be induced by EV71 and Coxsackie A 16 viruses, only the former can induce brain encephalitis [21] and high mortality [22]. The average mortality of EV71 is in the range of 13% to 15.7% [23,24]. Neurological complications include the inflammation of the cerebral cortex, brain stem and spinal cord, which leads to aseptic meningitis, brain stem encephalitis, motor neuron death and acute flaccid paralysis [19,23,25]. EV71 can infect fibroblasts, epithelium, cells of the respiratory and gastrointestinal tracts and dendritic cells [19,21]. EV71 can evade the human immune surveillance system including both the systemic immune system and the innate immune system [21]. One of the mechanisms involves inhibiting the host cell interferon type I response through EV71 protein 3C, which enhances viral replication and promotes the pathological outcome of encephalitis [21]. EV71-infected lymphocytes and leucocytes bring EV71 across the blood-brain barrier and thus assist in the introduction of EV71 into the central nervous system [21] and promote the pathogenesis of encephalitis. EV71-induced brain stem encephalitis enhances catecholamine, cytokine and chemokine concentrations in vivo, which promote EV71-related immunopathogenesis [19]. Most of the severe symptoms occur in young children around 12 months old. Adults infected by EV71 are generally asymptomatic or present with mild symptoms [24]. Severe brain encephalitis may be caused by the evolution of EV71 [22,25,26,27]. However, there are still no effective antiviral treatments for EV71 infection. Therefore, it is urgent to develop an effective therapeutic agent or preventive vaccine.

SARS-CoV-2 (COVID-19) emerged in late 2019 and rapidly became a pandemic [28]. It is a single-stranded (+) RNA virus and a member of the beta-coronavirus genus including SARS-CoV and MERS-CoV [29,30]. Mortality in SARS-CoV-2 is caused by hyper-inflammation of the lung, which subsequently leads to acute respiratory distress/failure (ARDS) [31]. Most COVID-19 patients with ARDS are characterized by markedly elevated cytokine levels including interleukin (IL)-2, IL-6, IL-7, granulocyte colony-stimulating factor (G-CSF), interferon-γ inducible protein 10 (CXCL10), monocyte chemoattractant protein 1 (CCL2), macrophage inflammatory protein 1-α (CCL3) and tumor necrosis factor-α (TNF-α) [32]. Unlike with SARS-CoV and MERS-CoV, corticosteroids exacerbate SARS-CoV-2-associated lung injury as this virus may drive immunosuppression [33]. SARS-CoV-2 utilizes the spike glycoprotein on the envelope to bind the cellular receptor ACE2 and TMPRSS2 for cell entry [34]. To prevent the overload of medical support, there is an urgent demand for effective therapeutic drugs and preventive vaccines [35]. Among the potential targets for the suppression of SARS-CoV-2, a few studies have investigated repurposing drugs or small molecules that inhibit ACE2 and TMPRSS2 [34,36]. However, these two receptors are detected in many organs and blocking ACE2 or TMPRSS2 may cause unwanted side effects particularly when potential antiviral agents are systemically administrated in patients with multiple comorbidities [37,38]. However, most known mechanisms of antiviral agents target a limited number of pathways, which may not be related to the pathophysiology of SARS-CoV-2 infection and such agents may cause intolerable adverse effects [39]. Concerning the safety of antiviral agents, the use of TCM warrants further exploration as various plant-derived compounds in TCM have been shown to exert minimal toxicities or have only mild side effects [40,41,42]. Although numerous complex TCM formulae have been investigated to determine their therapeutic effects against COVID-19, the findings of most such studies are largely based on patients’ self-reported symptoms and the mechanism of action is generally not discussed [43]. Despite the large number of patents related to plant-derived compounds with a putative therapeutic value in the treatment of SARS-CoV-2 [44], the use of structure-based screening remains a considerable challenge as the results of the aforementioned research efforts have not been functionally validated in vitro or in vivo [45]. Due to strict restrictions on the use of SARS-CoV-2 and the limited availability of suitable research facilities, it is difficult to directly investigate the activity of this virus in general laboratories.

Our previous study on the antiviral activity of honeysuckle revealed that inducing host innate miRNAs may have the potential to block the spread and infection of various viruses [2]. To extend this concept, herein we attempted to determine whether honeysuckle upregulated *let-7a* could target EV71 at predicted regions and alleviate its replication and pathogenesis. Taken together, our findings confirm the therapeutic effects of miRNA *let-7a* induced by honeysuckle aqueous extract on EV71 infection. Thus, we also predicate that honeysuckle-induced miRNAs including *let-7a* are potential antiviral agents against SARS-CoV-2 and may suppress the replication of SARS-CoV-2. Our encouraging findings warrant further investigation.

## 2. Materials and Methods

### 2.1. Preparation of Honeysuckle Aqueous Extract Preparation and Analysis of Blood miRNA Expression Profiles in Human Participants and Mice after Ingestion 

Dried honeysuckle flowers were purchased, identified and the fingerprint of compounds in the honeysuckle aqueous extracts was determined by UPLC-Q/TOF MS as described in the previous report [2]. A human volunteer assessment was conducted according to the Declaration of Helsinki and this study was approved by the Institutional Review Board (IRB) of National Cheng Kung University Hospital (IRB approval number IBR-99-127). Written informed consent was obtained from all participants. Animal welfare and experimental studies followed the guidelines of the Care and Use of Laboratory Animals (National Research Council, 1996) and this study was approved by the Institutional Animal Care and Use Committee (IACUC) of the Model Animal Research Center (MARC) of Nanjing University and the IACUC of National Cheng Kung University (approval number No. 101291). The experimental procedure on the human subjects and the mice as well as the analysis of blood miRNA profiles were described in detail in the previous report [2]. The miRBase website (http://www.mirbase.org, accessed on 1 May 2021) was used to search for human homologues of the endogenous matched miRNAs of the mice [46]. The miRNA sequences (from “miRBase 22 release”) and the viral sequences (SARS-COV-2: NCBI accession number NC045512; DENV2 PL046: NCBI accession number AJ968413; EV71 71-4643-TW98: NCBI accession number AF304458) were queried by “miranda v3.3a” to predict the miRNA target sequences of virus genomes using the following parameters: scaling parameter: 4; gap open penalty: −4; gap extension penalty: −3; score threshold: 140; minimum free energy threshold: −18. The family of clustering and seed sequence group of the miRNAs were analyzed using the “smirnaDB” platform (http://www.mirz.unibas.ch/cloningprofiles, accessed on 1 May 2021).

### 2.2. Prediction of Honeysuckle-Induced miRNA and Pathway Analysis

The proteins or genes targeted by the ingredients of honeysuckle were obtained from “SymMap” (https://www.symmap.org/, accessed on 1 May 2021), a comprehensive TCM database that integrates 499 TCM herbs registered in the Chinese pharmacopoeia with 19,595 ingredients and the corresponding 4302 genetic targets [47]. The genetic target values were filtered by different values of false discovery rate using the Benjamini–Hochberg procedure (FDR-BH). All significant genetic targets were used to query the miRNA of interest in our prediction list using the “g:Profiler” (https://biit.cs.ut.ee/gprofiler/, accessed on 1 May 2021) platform [48,49]. In this web-based tool, the statistical domain scope was set as “all known genes” and the significance threshold was set as “Benjamini–Hochberg FDR 0.05”.

Likewise, all data of interest pertaining to significant genetic targets can be used for pathway analysis by querying the free public online software “ConsensusPathDB” (CPDB) (http://cpdb.molgen.mpg.de/, accessed on 1 May 2021) or the commercial platform “Ingenuity Pathways Analysis” (IPA). CPDB provides information on integration networks in *Homo sapiens* including genetic, signaling, drug-target interactions and biochemical pathways. Data originate from 32 public resources and interactions that have been curated from the literature [50]. The parameters used for the over-representation analysis in CPDB include all 13 available databases and a cutoff *p*-value set as 0.05. IPA is a commercial web-based software package, which provides causal analysis approaches with a particular focus on the precise underlying upstream biological causes and probable downstream effects on cellular and organismal biology [51]. In the core analysis part of IPA, the cutoff *p*-value is set as 0.05.

### 2.3. Cell Culture and Viruses

A human rhabdomyosarcoma RD cell line (ATCC, CCL-136), a human neuroblastoma SK-N-SH cell line (ATCC, HTB-11) and a human kidney HEK 293T cell line (ATCC, CRL-11268) were cultured in Dulbecco’s modified Eagle’s medium (DMEM; GIBCO-BRL, Carlsbad, CA, USA) containing L-glutamine and 10% fetal bovine serum (FBS; Trace Bioscience, Sydney, Australia). Monkey kidney BT7-H cells (a gift from Stanley Lemon, University of Texas Medical Branch) were cultured in DMEM with 10% FBS and Geneticin™ (G-418 sulfate; Life Technologies, Grand Island, New York, USA). Cells were maintained at 37 °C in a 5% CO_2_ incubator.

The enterovirus 71 Tainan strain 4643/98 (EV71/4643; NCBI accession number AF304458) was propagated for one generation in RD cells before use. The mouse-adapted EV71 MP4 strain (EV71 MP4; NCBI accession number JN544419) [52,53] derived from the EV71/4643 strain was used to infect ICR mice (a gift from Dr. Chun-Keung Yu, National Cheng Kung University, Taiwan).

Human RD or SK-N-SH cells were cultured in a 6-well plate (3 × 10^5^ cells/well) overnight at 37 °C, followed by a replacement with a fresh DMEM medium containing 2% FBS in the presence or absence of EV71. The cells were maintained for the indicated times and harvested for analysis.

### 2.4. Plaque Assay

RD cells (2 × 10^5^ cells/well) were plated onto a 24-well plate (NUNC, Roskilde, Denmark) and placed at 37 °C for 16–20 hr. After removing the medium, cells were infected with the virus with DMEM containing 2% FBS (200 μL/well). After absorption at 37 °C for 1 hr, the viral suspension was replaced with a two-fold DMEM containing 2% FBS and 1% methyl cellulose solution (American Biorganics, Niagara Falls, NY, USA). The overlay medium was poured off at 72 hr post-infection (PI) and a 10% crystal violet solution was added for 15 min until it colored the cytoplasm. The excess was gently removed with water to show the uncolored location of dead cells (plaque). The viral titer was expressed as plaque-forming units per milliliter (pfu/mL).

### 2.5. Western Blot Analysis

At the indicated times after EV71 infection, the culture medium was removed and the cells in the 6 cm culture plates were washed twice with PBS followed by lysis with 50 μL of a radioimmunoprecipitation assay buffer (RIPA buffer; 10 μL of PMSF (0.1 M), 10 μL of aprotinin (2 mg/mL), 20 μL of EGTA (0.1 M), 5 μL of leupeptin (2 mg/mL) and 4 μL of sodium orthovanadate (Na_3_VO_4_, 0.5 M)). The cell lysates were harvested by centrifugation at 14,000 rpm at 4 °C for 10 min. The supernatants were collected and the protein was quantified by Bicinchoninic Acid (BCA) kits (Pierce, Rockford, IL, USA). The proteins were analyzed by the sodium dodecyl sulfate polyacrylamide gel electrophoresis (SDS-PAGE) followed by a transfer onto a polyvinylidene fluoride (PVDF) membrane (Millipore, Boston, MA, USA). The membrane was incubated with a TBST (tris-buffered saline with 0.1% Tween 20 detergent) blocking solution plus 5% non-fat dried milk for 1 hr followed by washing with TBST. The mouse monoclonal anti-EV71 VP2 antibody (1:2000 in TBST; Chemicon, Temecula, CA, USA) and mouse monoclonal anti-beta-actin antibody (1:5000 in TBST; Sigma, St. Louis, MO, USA) were used as the primary antibodies. After incubation at 4 °C overnight, the membranes were washed with TBST and followed by incubation with the secondary anti-mouse IgG antibody conjugated with horseradish peroxidase (1:5000; Chemicon, USA) for 1 hr at room temperature (RT). Finally, membranes were incubated with an Immobilon^TM^ Western Chemiluminescent HRP Substrate (Millipore) and examined using X-ray films. The protein level was measured and quantified using VisionWorks^TM^ LS image acquisition and analysis software (UVP, Upland, CA, USA).

### 2.6. Reporter Plasmids and Luciferase Assay

The wild-type and mutant sequences of EV71 5′UTR 233–254 and VP2 1234–1256 were cloned into Ambion^®^ pMIR-REPORT^TM^ miRNA Expression Reporter plasmid (Invitrogen) to form pMIR-5′UTR, pMIR-VP2 and their corresponding mutants.

Human HEK293T cells were co-transfected with miRNA *let-7a*, the reporter plasmid DNA of pMIR-5′UTR, pMIR-VP2 or their corresponding mutants and the internal control reporter plasmid pRL-TK Vector (Promega, Madison, WI, USA) by transient transfection using TurboFect^TM^ Transfection Reagent (Fermentas, Sankt Leon-Rot, Germany). The cells were harvested at 24 h for luciferase assay using the Dual-Luciferase^®^ Reporter Assay System (Promega). The luciferase activity was determined using a Mini-Lumat LB9506 luminometer (Berthold, Bad Wildbad, Germany). Each treatment was measured in triplicate and the experiment was performed three times independently.

### 2.7. RT-PCR and Real-Time PCR

EV71 positive-stranded (+) or negative-stranded (−) RNA was reversely transcribed into cDNA using High-Capacity cDNA Reverse Transcription Kits (Applied Biosystems, Foster City, CA, USA). To determine the level of (+) or (−) strand EV71 RNA, PCR was performed to evaluate the level of VP2 (as an indicator of EV71 RNA level) using a YEA DNA polymerase (Yeastern Biotech, Taipei, Taiwan). An NCode™ VILO™ miRNA cDNA Synthesis Kit (Invitrogen) was used to reversely transcribe miRNA *let-7a* into cDNA and NCode^TM^ and SYBR^TM^ Green miRNA qRT-PCR Kits (Invitrogen) were used for analysis. Real-time PCR was conducted using the Step One Real-Time PCR System (Applied Biosystems).

For the construction of the pMIR reporter plasmids, the primers used were as follows:

The wild-type 5′UTR of EV71 was 5′-AATGCACTAGTCGTTACCCGGACCAACTACTTCAGCTCAGCAAGCTTAATGC-3′.

The wild-type antisense of 5′UTR of EV71 was 5′-GCATTAAGCTTGCTGAGCTGAAGTAGTTGGTCCGGGTAACGACTAGTGCATT-3′.

The mutant 5′UTR of EV71 was 5′-AATGCACTAGTCCAATGCGGTCGATGAAGTACCGCTCAGCAAGCTTAATGC-3′.

The mutant antisense of 5′UTR of EV71 was 5′-GCATTAAGCTTGCTGAGCGGTACTTCATCGACCGCATTGGACTAGTGCATT-3′.

The wild-type VP2 of EV71 was 5′-AATGCACTAGTAAATGCACAGTTCCACTACCTCTAGCTCAGCAAGCTTAATGC-3′.

The wild-type antisense VP2 of EV71 was 5′-GCATTAAGCTTGCTGCGCCTAGAGGTAGTGGAACTGTGCATTTACTAGTGCAT-3′.

For the RT-PCR analysis, the primers used were as follows:

The negative strand (-) VP2 was 5′-GGCCACTCACCATAGCCAACTA-3′.

The (−) VP2 forward was 5′-ATCCGGGAATTTCCAGTACC-3′.

The (−) VP2 reverse was 5′-GCAAGAAGCAGCAAACATCA-3′.

For real-time PCR and RT-PCR, the primers used were as follows:

The VP2 forward was 5′-ACCCTTTGATTCTGCCTTGA-3′.

The VP2 reverse was 5′-CCTTGCGTAACTGCTTGTC-3′.

For endogenous β-actin mRNA, the primers used were as follows:

The β-actin-F was 5′-GGCGGCACCACCATGTACCCT-3′.

The β-actin-R was 5′-AGGGGCCGGACTCGTCATACT-3′.

For *let-7a*, the *snoRNA55* of mice and the *U54* of humans were as follows:

The hsa-*let-7a* was 5′-GCCTGAGGTAGTAGGTTGTATAGTTA-3′.

The snoRNA55 (Mus) was 5′-TGACGACTCCATGTGTCTGAGCAA-3′.

The U54 (homo) was 5′-GGTACCTATTGTGTTGAGTAACGGTGA-3′.

### 2.8. Suckling Mouse Model for EV71 Infection

To evaluate the effect of honeysuckle or *let-7a* on EV71 replication and related pathogenesis in vivo, the EV71 MP4 mouse-adapted strain and ICR suckling mice were used [54]. Despite that this suckling mouse model of EV71 infection could not present the clinical symptoms that mimicked infection in humans, it may serve as an indicative model for an evaluation of EV71 virulence, replication and pathogenesis. The 7-day-old ICR suckling mice were obtained from the Animal Research Center of National Cheng Kung University. The EV71 (MP4 strain) was intracranially (IC) inoculated into the ICR suckling mice (25 μL or 50 μL in 2.5 × 10^5^ pfu/mouse or 5 × 10^5^ pfu/mouse, respectively) using a 30-gauge needle and 2% FBS/DMEM was used for the mock infection.

Initially, the effect of honeysuckle ingestion on *let-7a* miRNA levels in the blood of the suckling mice was evaluated. For a mouse with a body weight of 4 g, a dose of 0.0008 g of honeysuckle in 40 mL of ddH_2_O was given using a 1 mL syringe. The control group of mice was fed boiled ddH_2_O. The 6-day-old ICR suckling mice were fed the honeysuckle aqueous extract twice a day for four consecutive days. The mice were then sacrificed followed by the collection of the brain tissues and blood samples. The investigation of the effects of *let-7a* on EV71 MP4-infected ICR suckling mice was conducted by intracranial inoculation using a micro-injection syringe.

EV71 MP4-infected mice with either honeysuckle or *let-7a* treatment were evaluated according to the following disease scores: 0: healthy; 1: ruffled hair, hunchbacked or reduced mobility; 2: wasting; 3: forelimb or hindlimb weakness; 4: forelimb or hindlimb paralysis; 5: death. The levels of viral genes and proteins as well as the titers in the mice brains were measured. For the collection of brain samples, the mice were anesthetized with pentobarbital sodium (5 mg/mouse; Nembutal; Abbott Lab., North Chicago, IL, USA) and then tissue samples were aseptically removed, weighed and homogenized either in 1 mL of 2% FBS/DMEM (for preparing the virus solution), 500 mL of TRIzol (for extracting the RNA) or 500 mL of a RIPA lysis buffer (for extracting protein).

## 3. Results

### 3.1. Honeysuckle-Induced Host miRNAs Both in Mice and Humans Were Predicted to Be Capable of Targeting Genome Sequences of SARS-CoV-2, DENV and EV71

**Figure 1:**
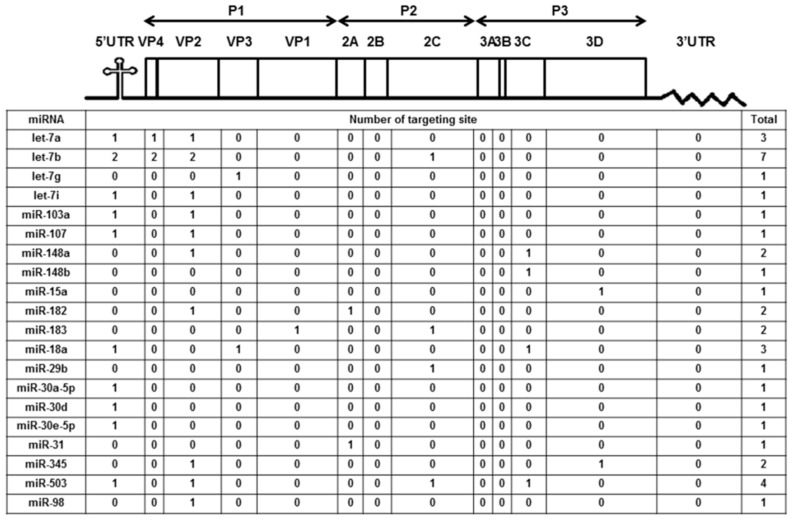
Honeysuckle ingestion induced twenty miRNAs both in mice and humans that are able to recognize the EV71 genome at various regions and the corresponding proteins. Four hundred 10-week-old C57/B6 mice and 17 healthy human volunteers ingested honeysuckle aqueous extract as described in the Methods. The upregulated endogenous miRNAs in the blood of the mice and humans were determined [2]. The overlapped miRNAs between mice and humans predicted to be able to target EV71 at multiple regions by “miranda v3.3a” are illustrated. The miRNA sequences were from “miRBase 22 release” and the viral sequences were from the Nucleotide database of NCBI (Accession No. AF304458).

We previously reported the differentially expressed miRNAs both in mice (10 weeks old) and human volunteers (23 to 30 years old) after ingesting honeysuckle for four consecutive days (twice a day) [2]. Among them, a total of 20 upregulated miRNAs both in mice and humans were predicted to be capable of targeting the EV71/4643 genome (Accession No. AF304458) at multiple regions (Figure 1). Moreover, 15 overlapping miRNAs in the mice and human groups were predicted to be able to target the genome sequences of SARS-CoV-2, DENV2 and EV71 (Supplementary Table S1). These data imply that the ingestion of honeysuckle both in mice and humans could induce a similar group of host miRNAs that have the potential to specifically target the genome of invading pathogens especially RNA viruses such as SARS-CoV-2, DENV-2 and EV71. To confirm that honeysuckle upregulated miRNA can indeed target these viruses and can attenuate viral replication, the effects of honeysuckle-mediated *let-7a* on EV71 infection both in vitro and in vivo was evaluated.

### 3.2. In Silico Prediction of Honeysuckle-Related Pathways Are Highly Correlated to Diverse Virus Infections and Regulation of Cytokines

The abovementioned 159 significant genes were used to query “CPDB” and “IPA” platforms for possible pathways. The canonical pathway analysis showed that honeysuckle participated in various viral infection mechanisms (Table 1 and Table 2) such as cytomegalovirus, Epstein–Barr virus, herpes virus, human T-cell leukemia virus 1, human immunodeficiency virus 1 infection, papillomavirus and Ebola virus. There was also a large number of cytokine-related pathways corresponding to the honeysuckle genetic targets especially IL-6, IL-8, IL-10 and IL-17, which were highly correlated to the cytokine storm in ARDS. In addition, the Ras activation signaling pathway was also targeted by honeysuckle, which was consistent with our previous finding [2].

### 3.3. *Let-7a* Bound with Two EV71 Replicon Clones and Attenuated Luciferase Activity

**Figure 2:**
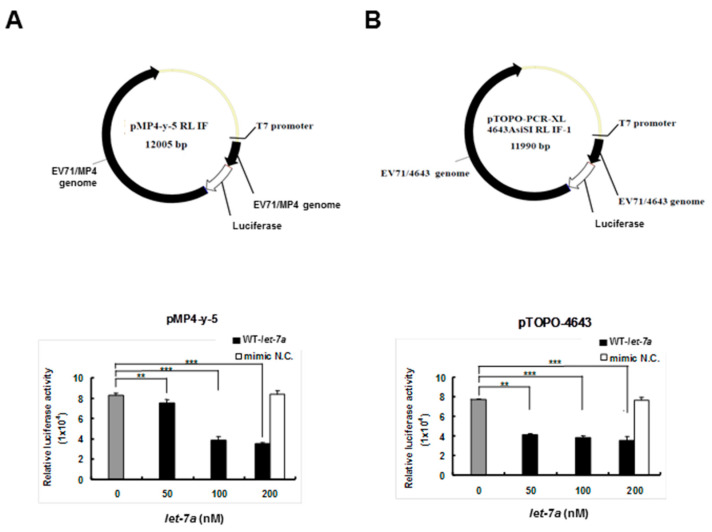
*Let-7a* suppressed the expression levels of 4643 and MP4 strain-infected EV71 clones in BT7-H cells. BT7-H cells were co-transfected with infectious clone plasmids (pMP4-y-5 and pTOPO-4643) and different concentrations of *let-7a* by TurboFect^TM^. Twenty-four hours after transfection, cells were harvested and protein lysate was subjected to the luciferase assay by a dual luciferase reporter assay system. The relative luciferase activity is shown in (**A**) for strain MP4 pMP4-y-5 and (**B**) strain 4643 pTOPO-4643. Mimic NC miRNA: scrambled sequence of *let-7a*. The relative luciferase activity was normalized with *Renilla* luciferase. **: *p* < 0.01, ***: *p* < 0.001

To clarify whether *let-7a* specifically targeted EV71 RNA genome sequences, two firefly luciferase reporter gene recombinant plasmids containing the genome replicon of either EV71 strain 4643 (pTOPO-4643) or mouse-adapted strain MP4 (pMP4-y-5P) (a gift from Dr. Jen-Ren Wang, National Cheng Kung University, Tainan, Taiwan) [55] were co-transfected together with the exogenous *let-7a* into BT7-H cells, which expressed T7 RNA polymerase to drive the viral gene expression of the recombinant plasmids. Twenty-four hours after transfection, the cellular protein was extracted and analyzed for dual luciferase activity using a luminometer. The results showed that the transfected *let-7a* significantly inhibited the luciferase activities of both pMP4-y-5p (Figure 2A) and pTOPO-4643 plasmids (Figure 2B) in a dose-dependent manner and the negative control miRNA (NC) showed no inhibition. This result implied that *let-7a* may target the RNA genome of the two EV71 strains.

### 3.4. *Let-7a* Was Confirmed to Specifically Target the EV71 Genome at Two Regions, 5′UTR 233–254 and VP2 1234–1256

**Figure 3:**
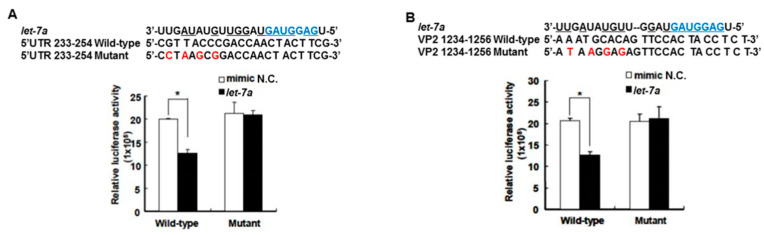
*Let-7a* specifically targeted 5′UTR and VP2 sequences of EV71. The predicted *let-7a* targeted EV71 sequences, 5′UTR 233–254 and VP2 1234–1256, and their mutants were cloned into the pMIR™ reporter plasmid. The reporter plasmid was then co-transfected with 200 nM *let-7a* into the HEK 293T cells. The luciferase activity of the reporter plasmid was evaluated at 24 h post-transfection. The suppressive effect and specificity of *let-7a* on 5′UTR sequence 233–254 (**A**) and VP2 sequence 1234–1256 (**B**) are shown. The seed sequence of *let-7a* is depicted in a blue color. The mutated nucleotides on the viral targeted sequences are shown in a red color. The pairing nucleotides between *let-7a* and the viral sequences are underlined. The negative control of miRNA is a scrambled sequence of *let-7a* (NC). Mean ± SD, *: *p* < 0.05.

The target gene predicted by “miranda v3.3a” software revealed that among the miRNAs induced by the honeysuckle aqueous extract, *let-7a* was capable of targeting EV71 genome at three regions, 5′UTR 233–254, VP4 814–836 and VP2 1234–1256 (Figure 1). A sequence alignment analysis revealed that the sequences of 5′UTR were highly conserved among several enterovirus strains (Supplementary Table S2) [56,57]. The alignments of the EV71 5′UTR 233–254 and VP2 1234–1256 regions with predicted *let-7a* targeting sequences are shown in Figure 3 and the targeting score was higher than that for EV71 VP4 814–836. To verify the targeting capability of *let-7a* on these two regions, the luciferase reporter plasmids harboring either wild-type or mutant sequences of EV71 5′UTR 233–254 and VP2 1234–1256 regions were constructed. Exogenous *let-7a* and the corresponding reporter plasmid were co-transfected into the human embryonic kidney cells 293T. The luciferase activity of the cells containing the wild-type EV71 5′UTR 233–254 or VP2 1234–1256 reporter plasmid was significantly suppressed in the presence of the exogenous *let-7a* compared with the negative control (mimic NC). In contrast, the luciferase activity of the cells containing the mutant EV71 5′UTR or VP2 reporter plasmid showed no difference between *let-7a* and the mimic NC group (Figure 3A,B). These data confirmed that *let-7a* could specifically bind and target the EV71 genome at the predicted 5′UTR 233–254 and VP2 1234–1256 regions.

### 3.5. *Let-7a* Suppressed EV71 RNA Expression and Viral Titer in Various Infected Cell Lines

**Figure 4:**
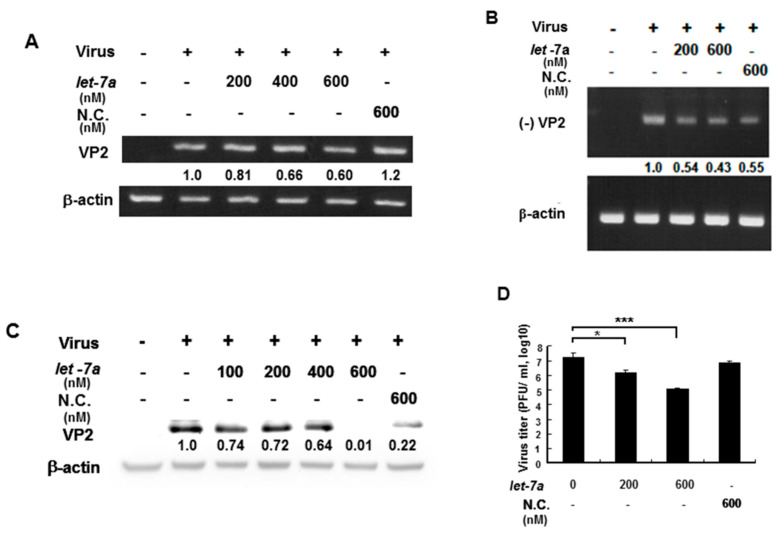
*Let-7a* suppressed EV71/4643 replication in infected RD cells. RD cells were transformed with *let-7a* for 24 hr before infection with EV71/4643 (MOI = 10) for 9 hr. (**A**) The suppression of *let-7a* at various concentrations on the viral RNA expression was evaluated by RT-PCR using the primers against (+) VP2 sequences. NC was the scramble control microRNA. The β-actin gene was used as the control. (**B**) The suppression of *let-7a* on negative-strand viral RNA expression (designated as (−) VP2) was evaluated by RT-PCR using the primers against (-) VP2 sequences. The negative control (NC) was the scramble control microRNA. The β-actin gene was used as the control. (**C**) *Let-7a* suppression of VP2 protein levels in viral-infected cells was determined by Western blotting using specific antibodies. β-actin was used as the internal control. (**D**) RD cells were transfected with *let-7a* or NC microRNA at various concentrations for 24 hr followed by infection with EV71/4643 (MOI = 10) for 1 hr. The virus titer was determined by plaque assay. Mean ± SD, *: *p* < 0.05, ***: *p* < 0.001.

EV71 contains a (+) single-stranded RNA genome, which is translated into a single polyprotein followed by proteolytically processing into mature single gene products. VP2 gene RNA and protein expression levels were measured by RT-PCR and Western blotting because *let-7a* specifically targets VP2 sequences so this was used to as an indicator to represent whether *let-7a* could suppress EV71 RNA and protein expression. An EV71/4643 strain was used to infect human muscle rhabdomyosarcoma RD cells in the presence or absence of exogenous *let-7a* by transient transfection. Our data showed that *let-7a* significantly decreased the levels of both the (+) stranded and (−) stranded RNA expression of the EV71 in a dose-dependent manner compared with infected cells without a *let-7a* transient transfection (Figure 4A,B). Similarly, *let-7a* inhibited the EV71 VP2 protein expression in the infected cells dose-dependently compared with the infected cells without a *let-7a* transient transfection (Figure 4C). Notably, a high concentration (600 nM) of non-specific scramble miRNA (NC) could also suppress viral (−) strained RNA and VP2 protein expression (Figure 4B,C) indicating that the non-specific scramble miRNA (NC) might target the viral (-) strained RNA and lead to the suppression of VP2 protein expression. Nevertheless, *let-7a* (600 nM) showed a much higher suppressive effect on VP2 protein expression (Figure 4C, lane 6 vs. lane 7). It indicated that the overexpression of *let-7a* could specifically suppress the replication of both progenitor and progeny viruses. Accordingly, the viral titer of EV71 was decreased in a *let-7a* dose-dependent manner in the infected RD cells compared with the infected cells without *let-7a* or with the scramble miRNA (NC) treatment by plaque assays (Figure 4D). Likewise, the effect of this specific suppression of *let-7a* on EV71 viral replication was also observed in EV71-infected neuronal SK-N-SH cells (Supplementary Figure S1). In summary, *let-7a* specifically suppressed EV71 RNA and protein expression and inhibited EV71 viral replication in various infected cells.

### 3.6. Honeysuckle Ingestion Attenuated Disease Symptoms, Prolonged Survival Time and Suppressed Viral Replication as Well as Viral Titer in EV71 MP4-Infected Suckling Mice

**Figure 5:**
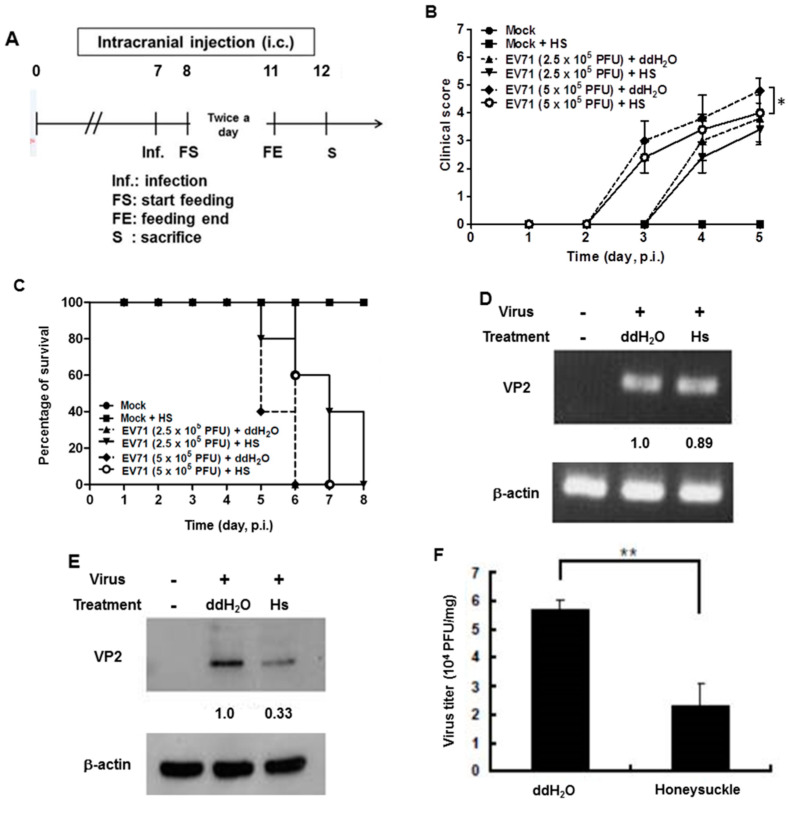
Honeysuckle treatment attenuated EV71/MP4 pathogenesis in infected ICR suckling mice. The 7-day-old ICR suckling mice (*n* = 5, each group) were infected with either a mock infection (2% FBS in DMEM) or EV71 MP4 by intracranial injection followed by treatment with honeysuckle aqueous extract twice a day for four consecutive days. Mice that received boiled distilled water served as the Mock control. The mice were then examined for survival rate, disease symptoms and the viral gene expression and production in the brain. (**A**) The time schedule of suckling mice with the virus infection, honeysuckle treatment and sacrifice. (**B**) The mice were intracranially inoculated (IC) with two concentrations of EV71/MP4 followed by honeysuckle treatment. The clinical scores were measured from day 1 to day 5 PI. (**C**) The survival time of the EV71/MP4 infected mice with or without honeysuckle treatment was evaluated for eight days after infection. The virus-infected suckling mice were sacrificed at day 5 PI. Viral activities in the brain specimens were investigated. (**D**) The EV71/MP4 RNA level in brains was determined by RT-PCR. β-actin was used as the internal control. (**E**) The EV71/MP4 VP2 protein level in the infected suckling mice brains was determined by Western blotting using specific antibodies. β-actin was used as the internal control. (**F**) The viral titer of EV71/MP4 in the infected suckling mice brains was determined by plaque assay. HS: honeysuckle; ddH_2_O: double distilled water; N = the number of mice in each group. Mean ± SD, *: *p* < 0.05, **: *p* < 0.01. Clinical score representation: 0: healthy; 1: ruffled hair, hunchbacked or reduced mobility; 2: wasting; 3: forelimb or hindlimb weakness; 4: forelimb or hindlimb paralysis; 5: death.

We previously reported that the *let-7a* expression level was strongly induced in the blood of 7-day-old ICR suckling mice after the ingestion of honeysuckle aqueous extract for four days [2]. To clarify whether honeysuckle or induced *let-7a* could suppress EV71 replication and affect the related pathogenesis in vivo, an ICR suckling mouse model was used [2]. The suppressive effect of honeysuckle on EV71 infection was evaluated using 7-day-old ICR suckling mice after an intracranial (IC) injection of an MP4 strain of EV71 followed by the ingestion of honeysuckle aqueous extract from day 8 to day 11 (two doses a day). All of the mice were sacrificed on day 12 (Figure 5A). The clinical scores representing disease symptoms of the EV71-infected mice were measured from day 2 PI. Two doses of EV71 were used in this study (2.5 × 10^5^ and 5 × 10^5^ pfu). Our data disclosed that clinical scores were detected on day 3 PI and honeysuckle treatment attenuated the clinical scores of the mice, which correlated with the titer of the infected virus (Figure 5B). Furthermore, the mice infected with viruses at either 2.5 × 10^5^ or 5 × 10^5^ pfu died on day 6 PI. Honeysuckle ingestion prolonged the survival time of the mice infected with viruses at 5 × 10^5^ or 2.5 × 10^5^ pfu for one to two days. No mice died without EV71 infection regardless of whether or not they ingested honeysuckle extract (Figure 5C). All of the mice in the Mock and Mock + HS groups remained normal (clinical score = 0) and showed a 100% survival rate.

We collected the brain tissues of EV71-infected mice receiving honeysuckle or ddH_2_O treatment at day 5 after virus inoculation and the viral RNA and protein levels as well as the virus titer were determined. We found that the viral RNA expression of EV71 was slightly decreased in the honeysuckle ingestion group (Hs) compared with the ddH_2_O group by RT-PCR analysis (Figure 5D). Notably, the VP2 protein expression was decreased by 67% in the honeysuckle ingestion group compared with the ddH_2_O group by Western blot analysis (Figure 5E). The virus titer in the brains of EV71-infected mice that ingested honeysuckle water was decreased by over 50% compared with the ddH_2_O group (Figure 5F). Taken together, our findings demonstrated that ingestion of honeysuckle one day after EV71 infection alleviated disease symptoms and extended the survival time of the infected suckling mice and these effects were accompanied by the suppression of EV71 replication as well as a decreased virus titer.

### 3.7. *Let-7a* Treatment Alleviated Clinical Scores, Prolonged Survival Time and Suppressed Viral Replication and Viral Titer in EV71 MP4-Infected Suckling Mice

**Figure 6:**
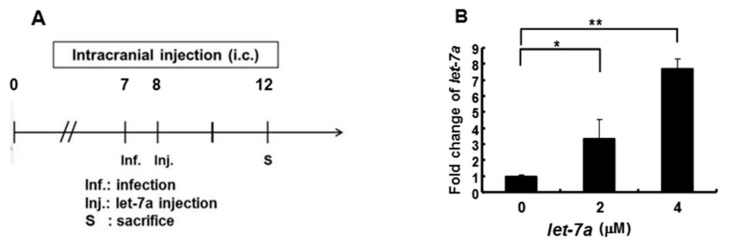

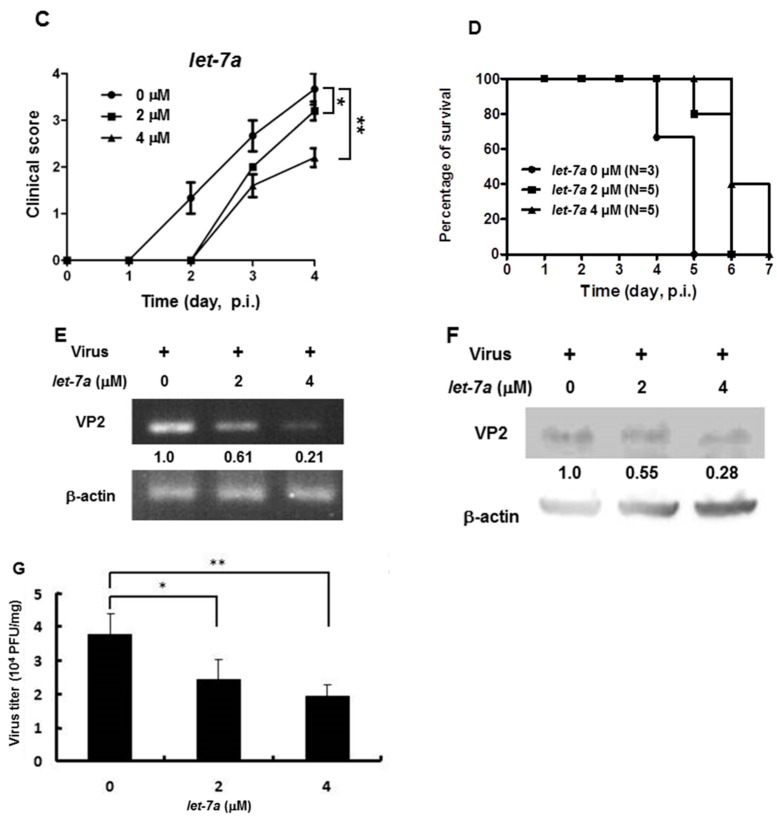
*Let-7a* treatment alleviated the pathogenesis of EV71/MP4-infected ICR suckling mice. Seven-day-old ICR suckling mice were inoculated IC with EV71 MP4 (5 × 10^5^ pfu per mouse). Indicated concentrations of *let-7a* were IC injected one day after virus inoculation. (**A**) The time schedule of the suckling mice with virus infection, *let-7a* injection and sacrifice. (**B**) Total RNA was harvested and the *let-7a* expression level in the brain tissues was determined by real-time PCR. (**C**) Clinical scores were measured daily after *let-7a* treatment. Mice brain tissues were collected four days after virus inoculation. (**D**) The EV71/MP4 VP2 protein level in the infected suckling mice brains with or without *let-7a* was determined by Western blotting using specific antibodies. β-actin was used as the internal control. (**E**) The expression level of VP2 was examined by specific primers and RT-PCR. The expression level of β-actin was used as an internal control. (**F**) Total protein was harvested and the expression level of the VP2 protein in the mice brain tissues was examined by Western blotting using specific antibodies. (**G**) The virus was harvested and the virus titer was determined by plaque assay. Mean ± SD, *: *p* < 0.05, **: *p* < 0.01.

As shown in Figure 2, Figure 3 and Figure 4, we demonstrated that *let-7a* could specifically target EV71-5′UPR and -VP2 sequences accompanied by the suppression of EV71 replication in infected cell lines. In Figure 5, it can be seen that honeysuckle ingestion alleviated the disease symptoms and prolonged the survival time of EV71-infected ICR suckling mice. *Let-7a* was the most upregulated miRNA induced by the ingestion of honeysuckle as detected both in human volunteers and in mice [2,20]. Therefore, we further clarified the effects of exogenous *let-7a* on the clinical scores and the survival time of EV71-infected ICR suckling mice and EV71 replication. The 7-day-old ICR suckling mice shown in Figure 5 were IC inoculated with EV71 MP4 (5 × 10^5^ pfu) at day 7 followed by an IC injection of *let-7a* (2 μM and 4 μM in 20 μL of DMEM) at day 8. All of the mice were sacrificed at day 12 (Figure 6A). The mice brains were sectioned and *let-7a* expression, virus protein, RNA and titer were measured. Our data showed that the levels of *let-7a* in the brains were elevated three-fold and seven-fold with a dose of 2 μM and 4 μM of *let-7a*, respectively (Figure 6B). Under these conditions, exogenous *let-7a* inoculation (2 and 4 μM) significantly attenuated the clinical scores of the EV71-infected mice on day 4 PI compared with the untreated EV71-infected mice (Figure 6C). Furthermore, *let-7a* inoculation at concentrations of 2μM and 4 μM prolonged the survival of the infected mice from day 5 to days 6 and 7, respectively (Figure 6D). Further studies revealed that *let-7a* treatment at concentrations of 2 μM and 4 μM resulted in a 39–79% reduction of viral RNA expression (Figure 6E) and a 45–72% reduction of VP2 protein expression (Figure 6F). Furthermore, inoculation with either 2 μM or 4 μM of *let-7a* decreased the viral titer in the brain tissues of the infected mice from 66% to 53% compared with the untreated control group (Figure 6G). In conclusion, *let-7a* inoculation showed a similar suppression effect on the uptake of honeysuckle including the alleviation of clinical scores and the extension of the survival time of EV71-infected mice. Furthermore, *let-7a* suppressed EV71 replication, leading to a decreased virus titer in the brains of the infected mice.

## 4. Discussion

The disease symptoms of EV71-infected human patients were defined as stage 1: hand, foot and mouth disease (HFMD) and herpangina; stage 2: CNS complication; stage 3: cardiopulmonary failure and pulmonary edema; stage 4: convalescence [23]. Accordingly, the clinical scores of EV71-infected suckling mice were classified as follows: score 0: healthy; score 1: ruffled hair, hunchbacked or reduced mobility; score 2: wasting; score 3: forelimb or hindlimb weakness; score 4: forelimb or hindlimb paralysis; score 5: death (see Materials and Methods). In our suckling mouse model, all of the infected mice died at day 5 or day 6 PI (Figure 5C and Figure 6D) indicating that EV71 intracranial infection is lethal to these mice. The pathogenesis of EV71-infected patients consists of direct damage to the target cells and organs as well as virus-induced immunopathogenesis including cytokine storm, inflammation, immune injury or cross-reactivity of antibodies to brain cells as well as antibody enhancement [58,59]. In this study, we found that either honeysuckle or *let-7a* could suppress EV71 VP2 gene expression and replication, which led to a decreased viral load accompanied by an attenuated pathogenesis and prolonged life span of the infected mice. These findings are similar to our previous report that showed either honeysuckle or *let-7a* could suppress DENV replication and pathogenesis. In addition, honeysuckle ingestion showed a preventive effect against the DENV2 challenge [2].

*Let-7a* targeted sequences on VP2 1234–1256 corresponding to EV71 amino acid sequences 163–171 (nucleotide sequence 1232–1258: caaaatgcacagttccactacctctat; peptide sequence 163–171: QNAQFHYLY; the major targeted nucleotide/peptide sequences are highlighted in bold; designated as: VP2 *let-7a* a163–171). VP2 is the capsid protein of EV71. VP2 together with VP1 can interact with the host cell receptor SCARB2 to provide attachment to the targeted cells [60]. Therefore, the finding that *let-7a* targeted the EV71 VP2 gene and reduced its expression implied that honeysuckle suppressed EV71 replication possibly by the blockage of the viral assembly and the prevention of viral maturation through the upregulation of endogenous *let-7a* expression.

The EV71 5′UTR region forms six stem-loop structures (from I to VI), which play important roles in viral replication and translation [17]. Furthermore, 5′UTR stem-loops II to VI contain the internal ribosome entry site (IRES) controlling the efficiency of translation initiation and viral replication during interactions with cellular factors [17,61]. Another *let-7a* targeting site on the EV71 genome is located at the 5′UTR 233–254 region, which encompasses the EV71 IRES sequence [17]. EV71 viral protein synthesis and IRES activity are up- or downregulated through interaction with the EV71 stem-loop IV by host factors PTB and FBP2, respectively [17]. Furthermore, host hnRNP K binds to the EV71 stem-loop IV to enhance viral RNA replication [17]. It is possible that honeysuckle-induced *let-7a* may interfere with the EV71 translation and replication by altering the stem-loop structure or interrupting the interaction between the host factors and the stem-loop IV. Moreover, *let-7a* maturation has been reported to be negatively regulated by a cellular factor hnRNP A1 to enhance the IRES activity [62,63].

*Let-7a* is a highly conserved miRNA, which was initially identified in *Caenorhabditis elegans* [64]. It is ubiquitously expressed in diverse tissues and organs [65,66,67]. *Let-7a* regulates development [64] and inhibits cell proliferation through the suppression of various target genes including proto-oncogene *Ras* [68,69], c-*Myc* [70,71], high mobility group protein (*HMGA2*) [72,73,74], *NIRF* [75], *E2F2* and *CCND2* [76]. It inhibits cell cycle and differentiation through the activation of p21WAF1 [75] and suppresses cell migration and apoptosis by targeting integrin beta-3 and caspase-3, respectively [77,78]. *Let-7a* is known as a tumor suppressor. The negative regulators of *let-7a* biogenesis including Lin28a, Lin28b and hnRNP A1 are associated with cancer formation [62]. We previously reported that honeysuckle treatment increased *let-7a* levels both in vitro and in vivo, which suppressed both DENV replication and the Ras protein level of the host cells [2]. These findings implied that honeysuckle-induced *let-7a* not only suppressed viral infection via the targeting of viral genome sequences but also led to the attenuation of tumor development by targeting the oncogene Ras.

Moreover, honeysuckle is one of the most commonly prescribed herbs in the treatment or prevention of COVID-19 according to an extensive analysis of the frequency of TCM used in China [79]. Honeysuckle was also predicted to be effective in treating SARS-CoV-2 infection. TCM formulae containing honeysuckle such as the Honeysuckle Decoction, Lianhuaqingwen capsules and the Shuang-Huang-Lian oral solution have been used as potential candidates for the treatment of SARS-CoV-2 infection [43]. The Honeysuckle Decoction inhibits SARS-CoV-2 replication and accelerates the recovery of infected patients [80]. Lianhuaqingwen capsules significantly inhibited the replication of SARS-CoV-2 and reduced IL-6, IL-8, CCL-2/MCP-1, CXCL-10 and TNF-α [81]. The Shuang-Huang-Lian oral solution also decreased the transcriptional and translational levels of TNF-α, IL-1β and IL-6 [82]. A randomized clinical trial (ChiCTR2000029822) conducted in China investigated the use of honeysuckle in the treatment of patients with COVID-19 and the results appeared to show a therapeutic benefit [80]. In addition, two reports disclosed that honeysuckle contains the small RNA designated as miRNA2911, which could specifically suppress the Varicella-zoster virus (VZV) and the influenza virus [83,84]. Taken together, the above findings indicate that honeysuckle per se and upregulated host miRNAs (Figure 1) including *let-7a* are capable of specifically targeting DENV and EV71 as well as the notorious SARS-CoV-2 (Supplementary Table S1) based on experimental evidence and in silico predication. In addition, we have revealed that honeysuckle is a broad spectrum antiviral agent because it induces multiple miRNAs that may target various viruses.

In our study, a pathway analysis using CPDB and IPA revealed a broad spectrum of the possible antiviral activity of honeysuckle (Table 1 and Table 2). A fulminant cytokine storm is the leading cause of mortality in COVID-19-induced ARDS [85]. Our analysis showed honeysuckle participated in the regulation of the majority of cytokines such as IL-2, IL-7 and TNF-α [32]. The miRNAs corresponding to the biological pathways of honeysuckle could be discovered using the “g:Profiler”-linked miRTarBase. The novel findings presented herein warrant further and more rigorous clinical trials to validate the use of honeysuckle in treating and preventing SARS-CoV-2 infection.

MiRNAs play multiple functions under different conditions. Thus, in order to develop a drug capable of inducing endogenous miRNAs that can target the viral genome and inhibit viral replication, we also need to consider its side effects on the host. Some miRNAs show a suppressive effect on viral replication but are also toxic to the host when they are highly expressed. In comparison, other naturally occurring products capable of suppressing EV71 activity may have stronger side effects in patients such as the famously toxic plant *Daphne Genkwa* that may cause circulatory arrest and internal bleeding [86] or *Rheum palmatum* that induces gastroenteritis and diarrhea [87,88]. Honeysuckle is a traditional herb that has long been used to treat diverse diseases including flu-like symptoms in China and is not known to induce any side effects. Nonetheless, further research is needed to establish the toxicity of honeysuckle extract in human subjects.

Our data showed that honeysuckle induced multiple innate miRNAs that might target SARS-CoV-2 and DENV2 as well as EV71 simultaneously (Supplementary Table S1). Furthermore, these miRNAs may recognize the viral genomes at multiple regions (Supplementary Table S1). Therefore, honeysuckle offers a unique advantage in that it can prevent drug resistance caused by viral RNA sequence mutation, a phenomenon seen with many antiviral drugs, by targeting multiple regions of the viral genome. Various active ingredients extracted from the honeysuckle plant have been reported to exhibit antimicrobial activities; however, the functional component in honeysuckle responsible for *let-7a* overexpression and its anti-EV71 activity remains incompletely understood. Furthermore, we cannot exclude the possibility that honeysuckle ingestion increased innate immunity through up-regulation of *let-7a* together with other active ingredients that may have contributed to the suppression of viral replication (SARS-CoV-2, DENV-2 and EV71) in the infected mice. Given that EV71 can evade human immune surveillance [21] and that vaccination may cause EV71-associated antibody enhancement, our promising findings may be of value in the development of a novel anti-EV71 treatment in combination with other symptom-relieving drugs. Most importantly, there is an urgent need to confirm that the modulation of the innate miRNA system using honeysuckle could contribute to the suppression of SARS-CoV-2.

## Figures and Tables

**Table 1:** The canonical pathway analysis by ConsensusPathDB (CPDB) ^§^.

Enriched Pathway-Based Sets	*q*-Value #	Source
Interleukin-4 and Interleukin-13 signaling	4.82E-29	Wikipathways
Human cytomegalovirus infection	2.40E-22	KEGG
Kaposi sarcoma-associated herpesvirus infection	6.30E-20	KEGG
Human T-cell leukemia virus 1 infection	1.94E-18	KEGG
Epstein–Barr virus infection	2.43E-18	KEGG
IL-17 signaling pathway	4.20E-17	KEGG
IL-2 receptor beta chain in t cell activation	4.41E-12	BioCarta
Validated transcriptional targets of AP1 family members Fra1 and Fra2	5.32E-12	PID
Human immunodeficiency virus 1 infection	8.74E-11	KEGG
Human papillomavirus infection	1.41E-10	KEGG
IL-2-mediated signaling events	3.35E-10	PID
IL-2 Signaling Pathway	4.95E-10	Wikipathways
Interleukin-10 signaling	4.18E-09	Wikipathways
IL-5 Signaling Pathway	6.59E-09	Wikipathways
IL-6	8.49E-09	NetPath
IL-3 Signaling Pathway	3.56E-08	Wikipathways
IL-11	4.30E-08	NetPath
IL-23-mediated signaling events	6.36E-08	PID
IL-7 Signaling Pathway	7.65E-08	Wikipathways
IL-4 Signaling Pathway	7.99E-08	Wikipathways
Interleukin-11 Signaling Pathway	2.44E-07	Wikipathways
IL-2 signaling events mediated by STAT5	2.80E-07	PID
IL-6-mediated signaling events	3.97E-07	PID
Regulation of Telomerase	5.31E-07	PID
IL-1	1.08E-06	NetPath
IL-2 signaling events mediated by PI3K	1.11E-06	PID
Signal transduction through il1r	1.11E-06	BioCarta
IL-1 signaling pathway	1.22E-06	Wikipathways
IL-2	1.29E-06	NetPath
Ras signaling in the CD4+ TCR pathway	1.91E-06	PID
IL-27-mediated signaling events	2.20E-06	PID
Ras signaling pathway	2.82E-06	KEGG
Fc-epsilon receptor I signaling in mast cells	2.83E-06	PID
IL-4	3.16E-06	NetPath
Human cytomegalovirus and map kinase pathways	3.74E-06	BioCarta
Ras Signaling	7.25E-06	Wikipathways
Signaling by Interleukins	7.27E-06	Reactome
Natural killer cell-mediated cytotoxicity	1.17E-05	KEGG
Activation of the AP-1 family of transcription factors	1.39E-05	Reactome
IL-6 signaling pathway	3.54E-05	Wikipathways
IL-4-mediated signaling events	3.66E-05	PID
IL-12-mediated signaling events	3.66E-05	PID
IL-7 signaling	3.76E-05	INOH
IL-10 anti-inflammatory signaling pathway	4.00E-05	BioCarta
Fc epsilon RI signaling pathway	4.30E-05	KEGG
IL8- and CXCR1-mediated signaling events	5.36E-05	PID
Fc epsilon receptor i signaling in mast cells	6.24E-05	BioCarta
Nfkb activation by nontypeable hemophilus influenzae	6.24E-05	BioCarta
IL-3	8.49E-05	NetPath
IL-12 signaling mediated by STAT4	9.82E-05	PID
IL-8- and CXCR2-mediated signaling events	0.000129	PID
West Nile virus	0.000231	BioCarta
MAPK family signaling cascades	0.000287	Reactome
Ccr3 signaling in eosinophils	0.000346	BioCarta
Ebola Virus Pathway on Host	0.00037	Wikipathways
Structural Pathway of Interleukin 1 (IL-1)	0.000392	Wikipathways
IL-1 and megakaryocytes in obesity	0.0004	Wikipathways
Signaling pathway from g-protein families	0.000528	BioCarta
IL-5	0.000562	NetPath
IL-7	0.000607	NetPath
IL-17 signaling pathway	0.000985	Wikipathways
Transcriptional regulation by the AP-2 (TFAP2) family of transcription factors	0.00113	Wikipathways
Regulation of Ras family activation	0.00123	PID
Calcium signaling by hbx of hepatitis b virus	0.00162	BioCarta
IL-1-mediated signaling events	0.00162	PID
IL-1 NFkB	0.00181	INOH
IL-9 Signaling Pathway	0.00187	Wikipathways
Transcriptional regulation by the AP-2 (TFAP2) family of transcription factors	0.00228	Reactome
TFAP2 (AP-2) family regulates transcription of cell cycle factors	0.00246	Reactome
Ras signaling pathway	0.0028	BioCarta
Fc epsilon receptor (FCERI) signaling	0.00348	Reactome
Interleukin-1 processing	0.00467	Reactome
FoxO family signaling	0.00467	PID

§ Date of access CPDB: 26 July 2020. # Significance was defined as a *q*-value < 0.01. CPDB prediction revealed that honeysuckle genetic target-related canonical pathways were involved in various viral infections such as the cytomegalovirus, Epstein–Barr virus, herpes virus, human T-cell leukemia virus 1, human immunodeficiency virus 1 infection, papillomavirus and Ebola virus. There were also a large number of cytokine-related signaling pathways corresponding to the honeysuckle genetic targets.

**Table 2:** The canonical pathway analysis by Ingenuity Pathways Analysis (IPA) ^§^.

IPA Canonical Pathways	−log (*p*-Value)
IL-12 signaling and production in macrophages	17.4
IL-8 signaling	14.8
IL-6 signaling	12.7
Role of PKR in interferon induction and antiviral response	12.2
IL-10 signaling	12
Role of pattern recognition receptors in recognition of bacteria and viruses	12
IL-7 signaling pathway	10.1
TNFR1 signaling	9.79
ILK signaling	9.45
IL-17A signaling in fibroblasts	8.9
IL-3 signaling	8.23
TNFR2 signaling	7.5
NF-κB activation by viruses	6.61
Differential regulation of cytokine production in macrophages and T helper cells by IL-17A and IL-17F	6.55
IL-23 signaling pathway	6.55
Differential regulation of cytokine production in intestinal epithelial cells by IL-17A and IL-17F	6.1
IL-15 signaling	5.5
IL-17 signaling	5.25
Regulation of IL-2 expression in activated and anergic T lymphocytes	5.04
IL-17A signaling in airway cells	4.28
LPS/IL-1-mediated inhibition of RXR function	4.25
IL-1 signaling	3.69
Mechanisms of viral exit from host cells	3.52
Natural killer cell signaling	3.27
IL-2 signaling	3.01
Virus entry via endocytic pathways	2.31
IL-9 signaling	2.3
IL-15 production	2.19
Role of RIG1-like receptors in antiviral innate immunity	2.1
IL-4 signaling	1.55

§ IPA date of access: 26 July 2020. Significance was defined as a –log (*p*-value) > 1.3. Pathway analysis by IPA showed honeysuckle affected the cytokine regulation such as altering the levels of IL-1, IL-10, IL-12, IL-8, IL-6 and TNF-α, which corresponded with bacterial or viral infection mechanisms.

## Data Availability

All data used in this study are already provided in the manuscript at the required section. There are no underlying data available.

## Supplementary Materials

The following are available online at https://www.mdpi.com/1999-4915/13/2/308/s1, Figure S1: Overexpressed *let-7a* in infected SK-N-SH cells inhibited EV71 replication, Table S1: The prediction of miRNA-§targeted sequences in SARS-CoV-2, DENV2 and EV71 genomes. Table S2: The sequence alignments of *let-7a* targeted EV71-5′UTR, VP2 and VP4 in various EV71 genomes.
