# Supplementary material for: Honeysuckle Aqueous Extracts Induced let-7a Suppress EV71 Replication and Pathogenesis In Vitro and In Vivo and Is Predicted to Inhibit SARS-CoV-2
Source: Viruses. 2021 Feb 16;13(2):308. doi: 10.3390/v13020308 (PMC7920029; doi:10.3390/v13020308)
Supplement: Supplementary file 1 [file viruses-13-00308-s001.pdf]

Supplementary Table 1. The prediction of miRNA-§targeted sequences in SARS-CoV-2, DENV2, and EV71 genome.

Unique miRNAs name	SARS-CoV-2 (#NC045512) targeted sequence and corresponding protein	DENV2 (#AJ968413) targeted sequence and corresponding protein	EV71 (#AF304458) targeted sequence and corresponding protein
let-7a§, #	4327-4348 (ORF1ab), 12121-12147 (ORF1ab), 28125-28150 (ORF8)	3310-3333 (NS1)	233-254 (5'UTR), 814-836 (VP4), 1234-1256 (VP2)
let-7b§, #	4329-4348 (ORF1ab), 10017-10042 (ORF1ab), 28587-28613 (N)	3314-3333 (NS1), 10639-10660 (3'UTR)	233-254 (5'UTR), 529-553 (5′UTR), 777-799 (VP4), 811-836 (VP4), 1234-1256 (VP2), 1536-1561 (VP2), 4526-4554 (2C)
let-7c	4328-4348 (ORF1ab), 12120-12147 (ORF1ab), 25496-25520 (ORF3a), 28590-28613 (N)	3314-3333 (NS1), 10639-10660 (3'UTR)	230-254 (5'UTR), 811-836 (VP4), 1232-1256 (VP2), 1536-1561 (VP2)
let-7d	833-858 (ORF1ab), 3436-3461 (ORF1ab), 9843-9867 (ORF1ab), 12121-12147 (ORF1ab), 17109-17139 (ORF1ab)	3311-3333 (NS1)	233-254 (5'UTR), 1232-1256 (VP2)
let-7f	4327-4348 (ORF1ab), 28125-28150 (ORF8)		1234-1256 (VP2)
let-7g§	9843-9867 (ORF1ab), 10163-10192 (ORF1ab), 28592-28613 (N)		2265-2287 (VP3)
let-7i§, #	3681-3703 (ORF1ab), 9845-9867 (ORF1ab), 28588-28613 (N)	10326-10349 (3'UTR)	532-553 (5′UTR), 1233-1256 (VP2)
miR-103a§, #	11584-11611 (ORF1ab), 12048-12072 (ORF1ab), 13070-13096 (ORF1ab), 14548-14568 (ORF1ab), 14565-14589 (ORF1ab), 14630-14658 (ORF1ab), 14762-14787 (ORF1ab), 15707-15729 (ORF1ab),	6445-6470 (NS4A)	341-367 (5′UTR), 1433-1460 (VP2)

	16567-16590 (ORF1ab),<br>16685-16712 (ORF1ab),<br>17948-17970 (ORF1ab),<br>19836-19863 (ORF1ab),<br>24076-24100 (S),<br>24215-24241 (S),<br>28717-28741 (N),<br>28899-28927 (N)		
miR-107§		109-212 (C),<br>6445-6470 (NS4A),<br>6614-6637 (NS4A)	341-367 (5′UTR),<br>1433-1460 (VP2)
miR-122	1605-1629 (ORF1ab),<br>2694-2712 (ORF1ab),<br>4872-4899 (ORF1ab),<br>5292-5316 (ORF1ab),<br>8943-8962 (ORF1ab),<br>17058-17078 (ORF1ab),<br>19305-19326 (ORF1ab),<br>21201-21220 (ORF1ab),<br>27721-27743 (ORF7a)	1198-1222 (E),<br>1342-1367 (E),<br>1817-1837 (E),<br>2632-2658 (NS1),<br>3784-3809 (NS2A)	1300-1320 (VP2),<br>2556-2583 (VP1),<br>4514-4534 (2C),<br>6843-6867 (3D)
miR-132	41-64 (5'UTR),<br>5544-5570 (ORF1ab),<br>7456-7476 (ORF1ab),<br>8323-8349 (ORF1ab),<br>22410-22437 (S)	1239-1260 (E),<br>3266-3296 (NS1),<br>8824-8848 (NS5)	619-644 (5′UTR),<br>1976-2003 (VP3),<br>3991-4012 (2B)
miR-133a	10632-10650 (ORF1ab)	7833-7853 (NS5),<br>7872-7853 (NS5)	1492-1515 (VP2),<br>5378-5396 (3B-3C),<br>5843-5872 (3C)
miR-140	10989-11010 (ORF1ab),<br>17864-17887 (ORF1ab),<br>24188-24212 (S),<br>27001-27030 (M)	6071-6095 (NS3)	5764-5786 (3C),<br>6534-6557 (3D)
miR-148a§, #	1287-1305 (ORF1ab),<br>8922-8943 (ORF1ab),<br>14029-14056 (ORF1ab),<br>22416-22443 (S),<br>28178-28199 (ORF8)	3074-3096 (NS1)	1262-1288 (VP2),<br>5890-5912 (3C)
miR-148b§	1287-1305 (ORF1ab),		5890-5912 (3C)

	2727-2754 (ORF1ab),<br>6329-6350 (ORF1ab),<br>14029-14056 (ORF1ab),<br>14197-14219 (ORF1ab),<br>17166-17188 (ORF1ab),<br>19081-19102 (ORF1ab)		
miR-15a§, #	6537-6560 (ORF1ab),<br>8445-8465 (ORF1ab),<br>8989-9017 (ORF1ab),<br>14545-14569 (ORF1ab),<br>16265-16291 (ORF1ab),<br>16297-16328 (ORF1ab),<br>19458-19490 (ORF1ab),<br>20672-20689 (ORF1ab),<br>22629-22652 (S)	5308-5331 (NS3)	6050-6071 (3D)
miR-15b	1560-1583 (ORF1ab),<br>6630-6653 (ORF1ab),<br>8599-8621 (ORF1ab),<br>16297-16328 (ORF1ab),<br>16693-16714 (ORF1ab),<br>16843-16864 (ORF1ab),<br>20671-20689 (ORF1ab),<br>22629-22652 (S),<br>24216-24243 (S),<br>24074-24101 (S),<br>29496-29520 (N)	6615-6639 (NS4A)	6047-6071 (3D),<br>6915-6938 (3D)
miR-182§, #	1488-1518 (ORF1ab),<br>13242-13266 (ORF1ab),<br>15663-15693 (ORF1ab),<br>19918-19947 (ORF1ab),<br>23618-23647 (S)	1573-1595 (E),<br>7003-7035 (4A)	1127-1158 (VP2),<br>3477-3509 (2A)
miR-183§, #	10421-10444 (ORF1ab),<br>12332-12352 (ORF1ab),<br>16942-16964 (ORF1ab),<br>17227-17254 (ORF1ab),<br>23732-23755 (S),<br>29259-29284 (N)	1568-1595 (E),<br>3153-3179 (NS1),4880-4899 (NS3)	3278-3310 (VP1),<br>4494-4516 (2C)
miR-184			438-458 (5'UTR)

miR-18a§, #	88-113 (5'UTR),<br>883-910 (ORF1ab),<br>5561-5588 (ORF1ab),<br>5650-5676 (ORF1ab),<br>9794-9814 (ORF1ab),<br>12880-12903 (ORF1ab),<br>19237-19262 (ORF1ab)	6671-6698 (NS4A)	50-73 (5'UTR), 2045-2071 (VP3), 5671-5695 (3C)
miR-195	7628-7649 (ORF1ab),<br>8995-9017 (ORF1ab),<br>22630-22652 (S),<br>26707-26730 (M)		5889-5909 (3C)
miR-199a		2378-2399 (E),<br>3387-3412 (NS1),<br>9908-9931 (NS5),<br>10615-10635 (3'UTR)	1132-1152 (VP2),<br>1332-1359 (VP2),<br>2534-2566 (VP1),<br>4649-4671 (2C),<br>5582-5613 (3C),<br>6663-6687 (3D)
miR-199b	553-580 (ORF1ab),<br>18062-18090 (ORF1ab)	1630-1649 (E),<br>10615-10635 (3'UTR)	1132-1152 (VP2),<br>4649-4671 (2C)
miR-204	3045-3070 (ORF1ab)	761-785 (M),<br>5570-5591 (NS3),<br>7865-7888 (NS5),<br>7991-8016 (NS5)	1349-1374 (VP2),<br>4471-4500 (2C),<br>4686-4709 (2C)
miR-206	4873-4899 (ORF1ab),<br>15677-15700 (ORF1ab),<br>28748-28777 (N)		1441-1465 (VP2)
miR-210	13431-13456 (ORF1ab),<br>17166-17188 (ORF1ab),<br>17447-17467 (ORF1ab),<br>23398-23417 (S)		1220-1243 (VP2),<br>2152-2178 (VP3),<br>6230-6251 (3D)
miR-211		5570-5591 (NS3),<br>7865-7888 (NS5)	1353-1374 (VP2),<br>4476-4500 (2C),<br>4689-4709 (2C)
miR-22	22320-22343 (S),<br>28848-28878 (N)		919-944 (VP4)
miR-24	945-973 (ORF1ab),<br>23209-23227 (S),<br>25326-25351 (S),		1715-1732 (VP3),<br>5235-5264 (3A)

	25679-25702 (ORF3a), 28598-28624 (N)		
miR-25	6436-6461 (ORF1ab), 17971-17996 (ORF1ab), 24518-24541 (S), 27144-27171 (M), 29747-29773 (3'UTR)		887-911 (VP4), 1272-1292 (VP2), 1949-1969 (VP3), 6266-6291 (3D)
miR-29b§, #	749-773 (ORF1ab), 2059-2081 (ORF1ab), 3712-3734 (ORF1ab), 4508-4526 (ORF1ab), 8768-8795 (ORF1ab), 11357-11381 (ORF1ab), 14873-14900 (ORF1ab), 16263-16291 (ORF1ab), 19461-19486 (ORF1ab), 19705-19735 (ORF1ab), 22327-22349 (S), 23041-23072 (S), 23645-23666 (S), 28625-28649 (N)	2391-2418 (E),3336-3359 (NS1), 5739-5767 (NS3), 6190-6212 (NS3), 10682-10706 (3'UTR)	4898-4923 (2C)
miR-30a-5p§, #	9640-9664 (ORF1ab), 20341-20366 (ORF1ab), 21974-21995 (S)	4619-4645 (NS3), 7944-7969 (NS5)	637-667 (5'UTR)
miR-30a-3p	16561-16582 (ORF1ab), 16599-16622 (ORF1ab)	4557-4579 (NS3), 5761-5783 (NS3), 8354-8375 (NS5), 9184-9205 (NS5), 10556-10580 (3’UTR)	4422-4443 (2C)
miR-30d§, #	8779-8803 (ORF1ab), 20341-20366 (ORF1ab), 21974-21995 (S)	4619-4645 (NS3)	637-667 (5'UTR)
miR-30e-5p§, #	4962-4981 (ORF1ab), 20341-20366 (ORF1ab), 21974-21995 (S), 29145-29168 (N)	4619-4645 (NS3)	637-667 (5'UTR)
miR-30e-3p	16599-16622 (ORF1ab), 18895-18918 (ORF1ab)	4555-4579 (NS3), 8353-8375 (NS5),	911-936 (VP4), 6068-6092 (3D)

		10562-10580 (3'UTR)	
miR-31§, #	5393-5414 (ORF1ab),<br>9424-9449 (ORF1ab),<br>23623-23645 (S),<br>28915-28934 (N)	3681-3706 (NS2A),<br>5623-5645 (NS3)	3382-3401 (2A)
miR-320a	6992-7015 (ORF1ab),<br>10027-10049 (ORF1ab),<br>10362-10390<br>(ORF1ab),<br>12111-12136 (ORF1ab),<br>12634-12658 (ORF1ab),<br>19310-19332 (ORF1ab),<br>22057-22087 (S)		2958-2980 (VP1),<br>3553-3575 (2A)
miR-324-5p	11222-11250 (ORF1ab),<br>15467-15495 (ORF1ab)	6050-6075 (NS3)	3638-3662 (2A),<br>5165-5192 (3A),<br>6214-6238 (3D),<br>6788-6801 (3D),<br>6905-6928 (3D)
miR-33			1880-1902 (VP3),<br>6277-6305 (3D)
miR-331		7435-7458 (NS4B),<br>7880-7898 (NS5)	2418-2445 (VP3),<br>3633-3657 (2A),<br>3689-3715 (2A)
miR-345§, #	22314-22334 (S)	9814-9835 (NS5)	1244-1268 (VP2),<br>6658-6681(3D)
miR-34a	3588-3609 (ORF1ab),<br>14568-14590 (ORF1ab),<br>23251-23273 (S),<br>26318-26341 (E),<br>28750-28777 (N)	1459-1480 (E),<br>3303-3331 (NS1)	312-337 (5'UTR)
miR-363	3019-3043 (ORF1ab),<br>5324-5345 (ORF1ab),<br>28414-28438 (N)	4528-4552 (NS3),<br>5080-5106 (NS3),<br>10408-10434 (3'UTR)	2885-2915 (VP1),<br>3682-3711 (2A)
miR-449	5272-5303 (ORF1ab),<br>17308-17328 (ORF1ab),<br>23620-23645 (S),<br>26321-26341 (E),<br>27537-27564 (ORF7a),	1454-1480 (E),<br>6500-6522 (NS4A)	315-337 (5'UTR),<br>5882-5909 (3C)

	29046-29066 (N)		
miR-451	14064-14092 (ORF1ab),<br>28552-28572 (N)	10483-10505 (3'UTR)	5867-5889 (3C)
miR-484		4802-4829 (NS3),<br>10243-10267 (NS5)	3998-4027 (2B),<br>5381-5402 (3B-3C)
miR-497	1562-1583 (ORF1ab),<br>6631-6653 (ORF1ab),<br>8442-8465 (ORF1ab),<br>8990-9017 (ORF1ab),<br>14546-14569 (ORF1ab),<br>16843-16864 (ORF1ab),<br>19469-19490 (ORF1ab),<br>22328-22352 (S),<br>22630-22652 (S),<br>28907-28928 (N),<br>29002-29030 (N)	6614-6639 (NS4A),<br>10636-10659 (3'UTR)	5358-5374 (3B)
miR-503§	3141-3170 (ORF1ab),<br>6988-7013 (ORF1ab),<br>10663-10688 (ORF1ab),<br>13708-13732 (ORF1ab),<br>14546-14569 (ORF1ab),<br>14564-14590 (ORF1ab),<br>14767-14788 (ORF1ab),<br>17149-17179 (ORF1ab),<br>19509-19531 (ORF1ab),<br>20913-20943 (ORF1ab),<br>23412-23432 (S),<br>24613-24641 (S),<br>25466-25489 (ORF3a),<br>25664-25687 (ORF3a),<br>26707-26730 (M),<br>28920-28945 (N),<br>28999-29030 (N)		197-218 (5'UTR),<br>1397-1418 (VP2),<br>4175-4199 (2C),<br>5888-5909 (3C)
miR-505	1878-1899 (ORF1ab),<br>2271-2296 (ORF1ab),<br>7978-8003 (ORF1ab),<br>8169-8195 (ORF1ab),	3263-3285 (NS1),<br>8824-8851 (NS5)	5132-5153 (3A),<br>6050-6070 (3D)

	22628-22652 (S)		
miR-92	24517-24541 (S)		2177-2202 (VP3),<br>3473-3503 (2A)
miR-93	881-910 (ORF1ab),<br>9211-9233 (ORF1ab),<br>15532-15563 (ORF1ab),<br>16951-16975 (ORF1ab),<br>17083-17110 (ORF1ab),<br>17449-17469 (ORF1ab),<br>27464-27485 (ORF7a)	8611-8633 (NS5)	157-178 (5'UTR), 526-553 (5’UTR), 3357-3377 (2A), 4547-4571 (2C)
miR-98§	12121-12147 (ORF1ab),<br>23437-23461 (S)		1232-1256 (VP2)

§: Honeysuckle ingestion upregulated miRNAs detected both in the young mice (10 weeks) and human volunteers

#: overlapping miRNAs between young mice and human groups in Figure 1 could target SARS-CoV-2, EV71, and DENV2

@ The miRNA sequences were from “miRBase 22 release” and the viral sequences were from the Nucleotide database of NCBI. The versions of COVID-19, DENV2, and EV A71 genome sequence were NC_045512.2, AJ968413.1, and AF304458.1, respectively. The miRNA target sites were predicted by miranda v3.3a using the following parameters: scaling parameter: 4; gap open penalty: -4; gap extension penalty: -3; score threshold: 140, and minimum free energy threshold: -18.

Supplementary Table 2. The sequence alignments of *let-7a*-targeted EV71-5′UTR, VP2 and VP4 in various strains of EV71 genomes.

Query	Subject	GenBank Title	percentage of identical matches	alignment length	start of alignment in query	end of alignment in query	start of alignment in subject	end of alignment in subject	expect value	bit score
let7a_AF304458_233-254	DQ060149.1	Human enterovirus 71 strain pinf7-54A from Taiwan, complete genome	100	22	1	22	233	254	0.001	34.6
let7a_AF304458_233-254	DQ381846.1	Human enterovirus 71 strain 6F/AUS/6/99, complete genome	100	22	1	22	232	253	0.001	34.6
let7a_AF304458_233-254	DQ341357.1	Human enterovirus 71 isolate 7F-AUS-6-99, complete genome	100	22	1	22	232	253	0.001	34.6
let7a_AF304458_233-254	FJ172159.1	Human enterovirus 71 isolate NUH0075/SIN/08, complete genome	100	22	1	22	193	214	0.001	34.6
let7a_AF304458_233-254	HQ424437.2	Enterovirus A71 strain 128135, complete genome	100	22	1	22	232	253	0.001	34.6
let7a_AF304458_233-254	HQ647178.1	Human enterovirus 71 isolate EV082_07, complete genome	100	22	1	22	230	251	0.001	34.6
let7a_AF304458_233-254	HQ647177.1	Human enterovirus 71 isolate EV073_07, complete genome	100	22	1	22	230	251	0.001	34.6
let7a_AF304458_233-254	HQ647176.1	Human enterovirus 71 isolate EV049_07, complete genome	100	22	1	22	229	250	0.001	34.6
let7a_AF304458_233-254	HQ647175.1	Human enterovirus 71 isolate EV053_07, complete genome	100	22	1	22	230	251	0.001	34.6

let7a_AF304458_233-254	HQ647173.1	Human enterovirus 71 isolate EV003_07, complete genome	100	22	1	22	230	251	0.001	34.6
let7a_AF304458_233-254	HQ647169.1	Human enterovirus 71 isolate EV090_07, complete genome	100	22	1	22	210	231	0.001	34.6
let7a_AF304458_233-254	HQ647167.1	Human enterovirus 71 isolate EV054_07, complete genome	100	22	1	22	231	252	0.001	34.6
let7a_AF304458_233-254	JN835312.1	Human enterovirus 71 strain MRS/09/3663, complete genome	100	22	1	22	232	253	0.001	34.6
let7a_AF304458_233-254	JN992285.1	Human enterovirus 71 strain 0964/SYD/98 CHO cell-adapted, complete genome	100	22	1	22	232	253	0.001	34.6
let7a_AF304458_233-254	JN992283.1	Human enterovirus 71 strain 0964/SYD/98, complete genome	100	22	1	22	232	253	0.001	34.6
let7a_AF304458_233-254	JX025559.1	Human enterovirus 71 strain LAZ60-TR, complete genome	100	22	1	22	232	253	0.001	34.6
let7a_AF304458_233-254	KC436270.1	Enterovirus A71 isolate V08-2236079, complete genome	100	22	1	22	232	253	0.001	34.6
let7a_AF304458_233-254	KP274876.1	Enterovirus A71 strain Taipei/C2-Y100/2011, complete genome	100	22	1	22	155	176	0.001	34.6
let7a_AF304458_233-254	KM077140.1	Enterovirus A71 strain Hau1, complete genome	100	22	1	22	233	254	0.001	34.6

let7a_AF304458_233-254	MG214681.1	Enterovirus A71 strain 30-2/2015/BJ, complete genome	100	22	1	22	232	253	0.001	34.6
let7a_AF304458_233-254	LC375764.1	Enterovirus A71 SI/Isehara/Japan/99 genomic RNA, complete genome	100	22	1	22	232	253	0.001	34.6
let7a_AF304458_233-254	MG672481.1	Enterovirus A71 isolate MRT-14-250, complete genome	100	22	1	22	224	245	0.001	34.6
let7a_AF304458_233-254	MG672480.1	Enterovirus A71 isolate SEN-14-157, complete genome	100	22	1	22	222	243	0.001	34.6
let7a_AF304458_233-254	MG672479.1	Enterovirus A71 isolate GIN-13-365, complete genome	100	22	1	22	232	253	0.001	34.6
let7a_AF304458_233-254	MG976582.1	Enterovirus A71 strain Taiwan/1998-20998, complete genome	100	22	1	22	145	166	0.001	34.6
let7a_AF304458_233-254	LR027543.1	Enterovirus A71 isolate STU546711, DEU08 genome assembly, complete genome: monopartite	100	22	1	22	232	253	0.001	34.6
let7a_AF304458_233-254	AF304458.1	Enterovirus 71 strain Tainan/4643/98 polyprotein mRNA, complete cds	100	22	1	22	233	254	0.001	34.6
let7a_AF304458_814-836	DQ060149.1	Human enterovirus 71 strain pinf7-54A from Taiwan, complete genome	100	23	1	23	814	836	4.59E-04	36.1
let7a_AF304458_814-836	KF982854.1	Enterovirus A71 isolate DL71, complete genome	100	23	1	23	815	837	4.59E-04	36.1

let7a_AF304458_814-836	KP274876.1	Enterovirus A71 strain Taipei/C2-Y100/2011, complete genome	100	23	1	23	737	759	4.59E-04	36.1
let7a_AF304458_814-836	KM077140.1	Enterovirus A71 strain Hau1, complete genome	100	23	1	23	814	836	4.59E-04	36.1
let7a_AF304458_814-836	MG976582.1	Enterovirus A71 strain Taiwan/1998-20998, complete genome	100	23	1	23	726	748	4.59E-04	36.1
let7a_AF304458_814-836	AF304458.1	Enterovirus 71 strain Tainan/4643/98 polyprotein mRNA, complete cds	100	23	1	23	814	836	4.59E-04	36.1
let7a_AF304458_1234-1256	DQ060149.1	Human enterovirus 71 strain pinf7-54A from Taiwan, complete genome	100	23	1	23	1234	1256	4.59E-04	36.1
let7a_AF304458_1234-1256	EU864507.1	Human enterovirus 71 isolate EV71/Zhejiang08, complete genome	100	23	1	23	1232	1254	4.59E-04	36.1
let7a_AF304458_1234-1256	KJ686180.1	Enterovirus A71 strain EV71/Homo sapiens/VNM/97/2012, complete genome	100	23	1	23	1211	1233	4.59E-04	36.1
let7a_AF304458_1234-1256	KP274876.1	Enterovirus A71 strain Taipei/C2-Y100/2011, complete genome	100	23	1	23	1157	1179	4.59E-04	36.1
let7a_AF304458_1234-1256	KP289430.1	Enterovirus A71 isolate EV71/P868/2013/China, complete genome	100	23	1	23	1231	1253	4.59E-04	36.1
let7a_AF304458_1234-1256	KM077140.1	Enterovirus A71 strain Hau1, complete genome	100	23	1	23	1234	1256	4.59E-04	36.1

let7a_AF304458_1234-1256	MG976582.1	Enterovirus A71 strain Taiwan/1998-20998, complete genome	100	23	1	23	1146	1168	4.59E-04	36.1
let7a_AF304458_1234-1256	AF304458.1	Enterovirus 71 strain Tainan/4643/98 polyprotein mRNA, complete cds	100	23	1	23	1234	1256	4.59E-04	36.1

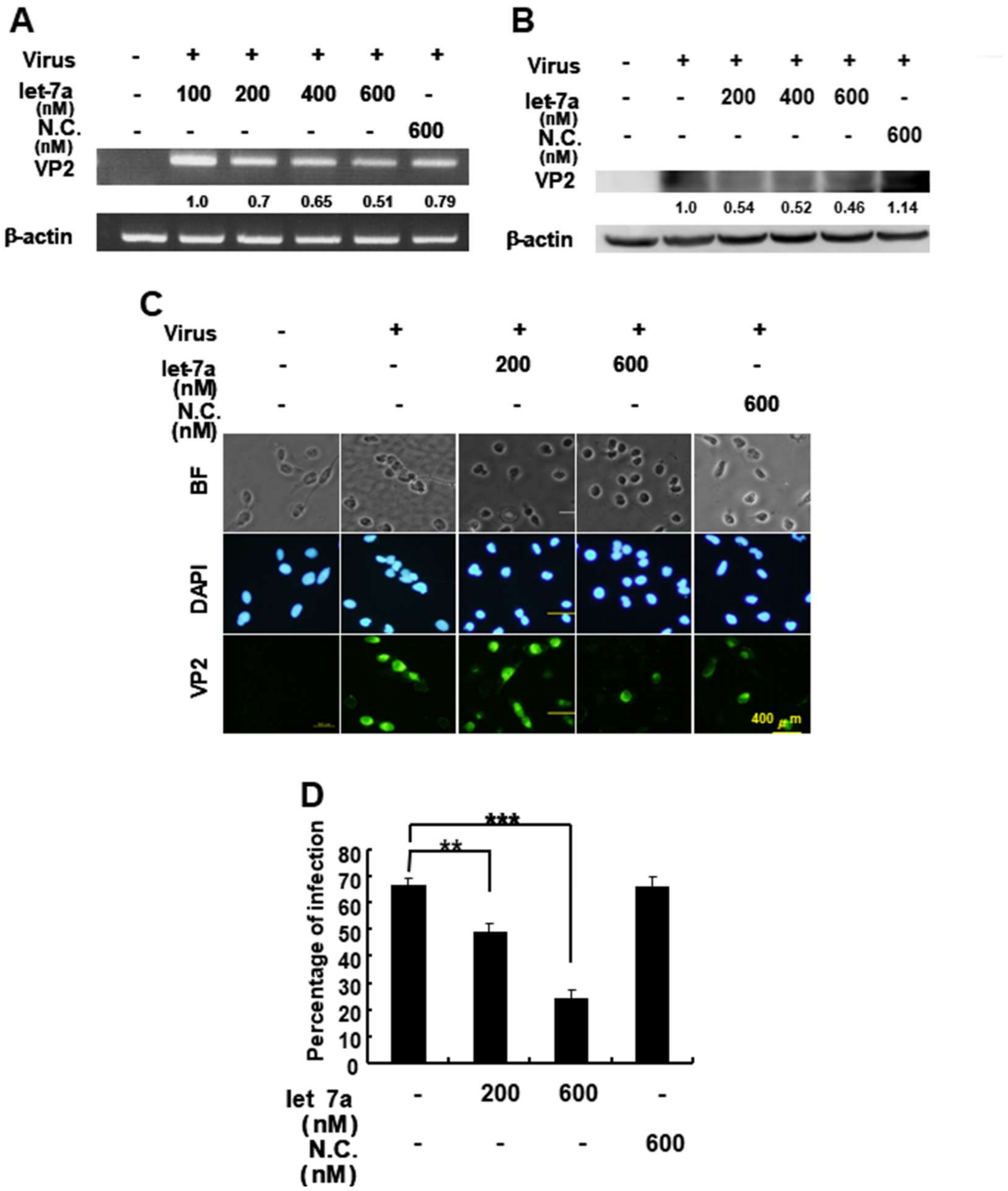

Supplementary Figure 1. Overexpressed *let-7a* in infected SK-N-SH cells inhibited EV71 replication.

Neuron SK-N-SH cells were transfected with different dosage of *let-7a* for 24 hr followed by infection with EV71MP4 for 9 hr at MOI of 10. (A) The total RNA of EV71-infected or uninfected cells in the presence of exogenous *let-7a* of various concentrations or scrambled microRNA (N.C.) was collected and the expression level of EV71 VP2 was evaluated by RT-PCR using VP2 specific primers. β-actin was used as the internal control. (B) The cell lysate was collected and the protein level of VP2 was determined by Western blotting. β-actin was used as the internal control. (C) The above cells after infection and treatment were fixed with 3.7% formaldehyde after 1x PBS wash followed by incubation with anti-EV71 VP2 monoclonal antibody

(green) and DAPI staining for nuclei overnight at 4ºC. The cells were investigated under a fluorescent microscope. BF: Bright field; Blue: nucleus (DAPI staining); Green: VP2 (D) The percentage of infected cells was quantified by random counting of five areas for the total cell number under a light microscope (BF) followed by counting of the green-labeled cells in fluorescent mode. The percentage is the average of the five random investigations. Values are the means ± SD. This experiment was repeated at least three times. Student's t test was used, **P<0.01, ***P<0.001 (compared to the value of mice not treated with *let-7a*).
